# (*E*)-2,4-Dihydr­oxy-*N*′-(2-hydr­oxy-3-meth­oxy-5-nitro­benzyl­idene)benzohydrazide dihydrate

**DOI:** 10.1107/S1600536810013000

**Published:** 2010-04-14

**Authors:** You-Yue Han, Yong-Hong Li, Qiu-Rong Zhao

**Affiliations:** a Department of Chemistry and Life Science, Chuzhou University, Chuzhou, Anhui 239000, People’s Republic of China

## Abstract

The asymmetric unit of the title compound, C_15_H_13_N_3_O_7_·2H_2_O, consists of a hydrazone mol­ecule and two solvent water mol­ecules. The mol­ecule adopts an *E* configuration with respect to the C=N bond. It is relatively planar, with a dihedral angle between the two benzene rings of 2.6 (1)°. There are intra­molecular O—H⋯N and O—H⋯O hydrogen bonds in the hydrazone mol­ecule. In the crystal structure, symmetry-related mol­ecules are linked through inter­molecular N—H⋯O and O—H⋯O hydrogen bonds to form a three-dimensional network.

## Related literature

For the biological properties of hydrazone compounds, see: Patil *et al.* (2010 ▶); Cukurovali *et al.* (2006 ▶). For the crystal structures of hydrazone compounds, see: Mohd Lair *et al.* (2009 ▶); Lin & Sang (2009 ▶); Suleiman Gwaram *et al.* (2010 ▶). For the hydrazone compounds we have reported on recently, see: Han & Zhao (2010*a* ▶,*b* ▶). For bond-length data, see: Allen *et al.* (1987 ▶). For the crystal structures of similar compounds, see: Li & Ban (2009 ▶); Lo & Ng (2009 ▶); Ning & Xu (2009 ▶); Zhu *et al.* (2009 ▶).
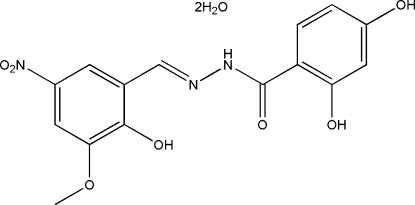


## Experimental

### 

#### Crystal data

C_15_H_13_N_3_O_7_·2H_2_O
*M* *_r_* = 383.32
Triclinic, 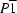

*a* = 7.976 (2) Å
*b* = 9.325 (2) Å
*c* = 11.547 (3) Å
α = 95.43 (2)°
β = 96.21 (2)°
γ = 102.01 (2)°
*V* = 829.0 (4) Å^3^
*Z* = 2
Mo *K*α radiation
μ = 0.13 mm^−1^
*T* = 298 K
0.23 × 0.20 × 0.20 mm

#### Data collection

Bruker SMART CCD area-detector diffractometer
Absorption correction: multi-scan (*SADABS*; Bruker, 2001 ▶) *T*_min_ = 0.971, *T*_max_ = 0.975
11747 measured reflections
4268 independent reflections
1790 reflections with *I* > 2σ(*I*)
*R*_int_ = 0.045

#### Refinement

*R*[*F* ^2^ > 2σ(*F* ^2^)] = 0.050
*wR*(*F* ^2^) = 0.143
*S* = 0.94
4268 reflections
263 parameters
7 restraints
H atoms treated by a mixture of independent and constrained refinement
Δρ_max_ = 0.16 e Å^−3^
Δρ_min_ = −0.23 e Å^−3^

### 

Data collection: *SMART* (Bruker, 2007 ▶); cell refinement: *SAINT* (Bruker, 2007 ▶); data reduction: *SAINT*; program(s) used to solve structure: *SHELXS97* (Sheldrick, 2008 ▶); program(s) used to refine structure: *SHELXL97* (Sheldrick, 2008 ▶); molecular graphics: *SHELXTL* (Sheldrick, 2008 ▶); software used to prepare material for publication: *SHELXTL*.

## Supplementary Material

Crystal structure: contains datablocks global, I. DOI: 10.1107/S1600536810013000/su2173sup1.cif

Structure factors: contains datablocks I. DOI: 10.1107/S1600536810013000/su2173Isup2.hkl

Additional supplementary materials:  crystallographic information; 3D view; checkCIF report

## Figures and Tables

**Table 1:** Hydrogen-bond geometry (Å, °)

*D*—H⋯*A*	*D*—H	H⋯*A*	*D*⋯*A*	*D*—H⋯*A*
O8—H8*B*⋯O4^i^	0.86 (2)	2.39 (2)	2.953 (2)	124 (2)
O8—H8*B*⋯O7^i^	0.86 (2)	2.18 (1)	3.001 (3)	163 (2)
O9—H9*B*⋯O3^ii^	0.85 (2)	2.19 (1)	3.032 (2)	170 (3)
O9—H9*A*⋯O2^iii^	0.86 (1)	1.98 (1)	2.840 (2)	176 (2)
O8—H8*A*⋯O9^iv^	0.86 (2)	1.93 (1)	2.786 (2)	176 (2)
N1—H1*A*⋯O5^v^	0.88 (1)	2.55 (2)	3.183 (3)	130 (2)
N1—H1*A*⋯O8^ii^	0.88 (1)	2.45 (2)	3.195 (3)	143 (2)
O4—H4⋯N2	0.82	1.85	2.569 (2)	146
O2—H2⋯O3	0.82	1.80	2.526 (2)	147
O1—H1⋯O8^vi^	0.82	1.91	2.718 (2)	169

Symmetry codes: (i) 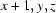; (ii) 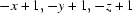; (iii) 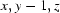; (iv) 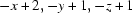; (v) 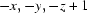; (vi) 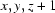.

**Fig. 1.**
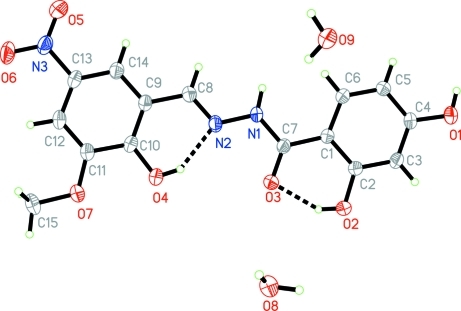
The molecular structure of the title compound, with atom labels and displacement ellipsoids drawn at the 30% probability level. Intramolecular O—H···O and O—H···N hydrogen bonds are shown as dashed lines.

**Fig. 2.**
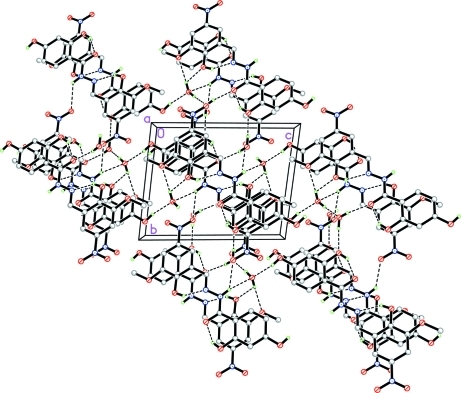
A perspective view, along the a axis, of the crystal packing of the title compound, showing the O—H···O, O—H···N, and N—H···O hydrogen bonds as dashed lines (see Table 1 for details).

## supplementary crystallographic information

### Comment

Hydrazone compounds have been widely investigated for their biological properties (Patil *et al.*, 2010; Cukurovali *et al.*, 2006). Furthermore, the crystal structures of the hydrazone compounds have also attracted much attention in recent years (Mohd Lair *et al.*, 2009; Lin & Sang, 2009; Suleiman Gwaram *et al.*, 2010). As a continuation of our work on the structural characterization of such compounds (Han & Zhao, 2010*a*,*b*), we report herein on the synthesis and crystal structure of the new title hydrazone compound.

The title compound, Fig. 1, consists of a hydrazone molecule and two water molecules of crystallization. The hydrazone molecule adopts an *E* configuration with respect to the C═N bond. The dihedral angle between the two benzene rings in the hydrazone molecule is 2.6 (1)°. There are intramolecular O—H···N and O—H···O hydrogen bonds in the hydrazone molecule (Fig. 1 and Table 1). All the bond lengths are within normal ranges (Allen *et al.*, 1987), and are comparable with those in similar compounds (Li & Ban, 2009; Lo & Ng, 2009; Ning & Xu, 2009; Zhu *et al.*, 2009).

In the crystal structure of the title compound symmetry related molecules are linked through intermolecular N—H···O and O—H···O hydrogen bonds to form a three-dimensional network (Table 1 and Fig. 2).

### Experimental

A mixture of 3-methoxy-5-nitrosalicylaldehyde (0.197 g, 1 mmol) and 2,4-dihydroxybenzohydrazide (0.168 g, 1 mmol) in 50 ml methanol was stirred at room temperature for 1 h. The mixture was then filtered to remove any impurities, and the filtrate left at room temperature for slow evaporation of the solvent. After a few days colourless block-like crystals of the title compound, suitable for X-ray diffraction, were formed.

### Refinement

Amino H and water H atoms were located from a difference Fourier map and refined isotropically, with N—H, O—H, and H···H distances restrained to 0.90 (1), 0.85 (1), and 1.37 (2) Å, respectively. The C-bound H atoms were positioned geometrically and refined using the riding-model approximation, with C—H = 0.93 and 0.96 Å for CH and methyl H atoms, respectively, O—H(hydroxyl) = 0.82 Å, with U_iso_(H) = k × U_eq_(parent C or O atom), where k = 1.2 for CH H atoms and k = 1.5 for O(hydroxyl) and methyl H atoms.

### Crystal data

**Table:**

C_15_H_13_N_3_O_7_·2H_2_O	*Z* = 2
*M**_r_* = 383.32	*F*(000) = 400
Triclinic, *P*1	*D*_x_ = 1.536 Mg m^−^^3^
Hall symbol: -P 1	Mo *K*α radiation, λ = 0.71073 Å
*a* = 7.976 (2) Å	Cell parameters from 1898 reflections
*b* = 9.325 (2) Å	θ = 2.6–25.6°
*c* = 11.547 (3) Å	µ = 0.13 mm^−^^1^
α = 95.43 (2)°	*T* = 298 K
β = 96.21 (2)°	Block, colourless
γ = 102.01 (2)°	0.23 × 0.20 × 0.20 mm
*V* = 829.0 (4) Å^3^	

### Data collection

**Table:**

Bruker SMART CCD area-detector diffractometer	4268 independent reflections
Radiation source: fine-focus sealed tube	1790 reflections with *I* > 2σ(*I*)
graphite	*R*_int_ = 0.045
ω scans	θ_max_ = 29.2°, θ_min_ = 1.8°
Absorption correction: multi-scan (*SADABS*; Bruker, 2001)	*h* = −10→10
*T*_min_ = 0.971, *T*_max_ = 0.975	*k* = −12→12
11747 measured reflections	*l* = −15→15

### Refinement

**Table:**

Refinement on *F*^2^	Primary atom site location: structure-invariant direct methods
Least-squares matrix: full	Secondary atom site location: difference Fourier map
*R*[*F*^2^ > 2σ(*F*^2^)] = 0.050	Hydrogen site location: inferred from neighbouring sites
*wR*(*F*^2^) = 0.143	H atoms treated by a mixture of independent and constrained refinement
*S* = 0.94	*w* = 1/[σ^2^(*F*_o_^2^) + (0.0611*P*)^2^] where *P* = (*F*_o_^2^ + 2*F*_c_^2^)/3
4268 reflections	(Δ/σ)_max_ = 0.001
263 parameters	Δρ_max_ = 0.16 e Å^−^^3^
7 restraints	Δρ_min_ = −0.23 e Å^−^^3^

### Special details

**Table:**

Geometry. All esds (except the esd in the dihedral angle between two l.s. planes) are estimated using the full covariance matrix. The cell esds are taken into account individually in the estimation of esds in distances, angles and torsion angles; correlations between esds in cell parameters are only used when they are defined by crystal symmetry. An approximate (isotropic) treatment of cell esds is used for estimating esds involving l.s. planes.
Refinement. Refinement of *F*^2^ against ALL reflections. The weighted *R*-factor wR and goodness of fit *S* are based on *F*^2^, conventional *R*-factors *R* are based on *F*, with *F* set to zero for negative *F*^2^. The threshold expression of *F*^2^ > σ(*F*^2^) is used only for calculating *R*-factors(gt) etc. and is not relevant to the choice of reflections for refinement. *R*-factors based on *F*^2^ are statistically about twice as large as those based on *F*, and *R*- factors based on ALL data will be even larger.

### Fractional atomic coordinates and isotropic or equivalent isotropic displacement parameters (Å^2^)

**Table:**

	*x*	*y*	*z*	*U*_iso_*/*U*_eq_	
O1	0.8600 (2)	0.84330 (17)	1.02241 (12)	0.0573 (5)	
H1	0.8627	0.7814	1.0681	0.086*	
O2	0.6371 (2)	0.89359 (17)	0.64100 (13)	0.0605 (5)	
H2	0.5636	0.8519	0.5863	0.091*	
O3	0.4100 (2)	0.68584 (17)	0.52377 (12)	0.0534 (4)	
O4	0.0820 (2)	0.45944 (17)	0.28656 (12)	0.0534 (4)	
H4	0.1460	0.4812	0.3491	0.080*	
O5	−0.3563 (2)	−0.1600 (2)	0.34555 (17)	0.0764 (6)	
O6	−0.4541 (2)	−0.1357 (2)	0.16969 (16)	0.0838 (6)	
O7	−0.1224 (2)	0.36004 (18)	0.09529 (12)	0.0555 (5)	
O8	0.8842 (2)	0.6698 (2)	0.19713 (15)	0.0631 (5)	
O9	0.8089 (2)	0.1954 (2)	0.65935 (17)	0.0796 (6)	
N1	0.3056 (2)	0.4776 (2)	0.59954 (14)	0.0453 (5)	
N2	0.1982 (2)	0.4267 (2)	0.49649 (14)	0.0441 (5)	
N3	−0.3569 (3)	−0.0901 (2)	0.26028 (19)	0.0568 (6)	
C1	0.5283 (3)	0.6659 (2)	0.71728 (17)	0.0384 (5)	
C2	0.6353 (3)	0.8076 (2)	0.72930 (18)	0.0423 (6)	
C3	0.7446 (3)	0.8642 (3)	0.83179 (19)	0.0472 (6)	
H3	0.8159	0.9579	0.8379	0.057*	
C4	0.7485 (3)	0.7825 (3)	0.92498 (17)	0.0427 (6)	
C5	0.6437 (3)	0.6424 (3)	0.91557 (18)	0.0466 (6)	
H5	0.6457	0.5871	0.9784	0.056*	
C6	0.5371 (3)	0.5857 (2)	0.81357 (18)	0.0455 (6)	
H6	0.4684	0.4909	0.8079	0.055*	
C7	0.4131 (3)	0.6119 (2)	0.60768 (17)	0.0390 (5)	
C8	0.0968 (3)	0.3000 (3)	0.48753 (18)	0.0464 (6)	
H8	0.0971	0.2439	0.5500	0.056*	
C9	−0.0187 (3)	0.2433 (2)	0.38010 (17)	0.0417 (5)	
C10	−0.0196 (3)	0.3255 (2)	0.28470 (18)	0.0419 (5)	
C11	−0.1331 (3)	0.2684 (3)	0.18101 (18)	0.0443 (6)	
C12	−0.2421 (3)	0.1325 (3)	0.17254 (19)	0.0481 (6)	
H12	−0.3166	0.0939	0.1040	0.058*	
C13	−0.2392 (3)	0.0535 (3)	0.26850 (19)	0.0471 (6)	
C14	−0.1305 (3)	0.1057 (3)	0.37055 (18)	0.0475 (6)	
H14	−0.1315	0.0496	0.4330	0.057*	
C15	−0.2229 (3)	0.3030 (3)	−0.01694 (19)	0.0638 (7)	
H15A	−0.1958	0.2112	−0.0447	0.096*	
H15B	−0.1962	0.3727	−0.0719	0.096*	
H15C	−0.3438	0.2869	−0.0090	0.096*	
H1A	0.296 (3)	0.418 (2)	0.6542 (17)	0.080*	
H8A	0.977 (2)	0.715 (2)	0.2410 (19)	0.080*	
H9A	0.759 (3)	0.1042 (12)	0.657 (2)	0.080*	
H9B	0.743 (3)	0.234 (2)	0.615 (2)	0.080*	
H8B	0.906 (3)	0.5873 (17)	0.172 (2)	0.080*	

### Atomic displacement parameters (Å^2^)

**Table:**

	*U*^11^	*U*^22^	*U*^33^	*U*^12^	*U*^13^	*U*^23^
O1	0.0587 (11)	0.0548 (11)	0.0468 (9)	−0.0006 (9)	−0.0125 (8)	−0.0006 (7)
O2	0.0653 (12)	0.0487 (10)	0.0564 (10)	−0.0081 (9)	−0.0119 (8)	0.0186 (8)
O3	0.0516 (10)	0.0577 (10)	0.0434 (9)	−0.0048 (8)	−0.0026 (7)	0.0162 (8)
O4	0.0512 (11)	0.0499 (10)	0.0491 (9)	−0.0038 (8)	−0.0075 (8)	0.0046 (8)
O5	0.0741 (14)	0.0609 (12)	0.0809 (13)	−0.0103 (10)	−0.0060 (10)	0.0162 (10)
O6	0.0693 (13)	0.0863 (14)	0.0677 (11)	−0.0289 (11)	−0.0142 (10)	−0.0032 (10)
O7	0.0570 (11)	0.0588 (11)	0.0426 (9)	0.0020 (8)	−0.0081 (7)	0.0056 (8)
O8	0.0602 (12)	0.0694 (13)	0.0548 (11)	0.0087 (10)	−0.0054 (8)	0.0095 (9)
O9	0.0685 (14)	0.0679 (14)	0.0864 (13)	−0.0128 (11)	−0.0181 (10)	0.0234 (11)
N1	0.0466 (12)	0.0447 (12)	0.0358 (10)	−0.0027 (9)	−0.0075 (9)	0.0036 (8)
N2	0.0404 (12)	0.0486 (12)	0.0382 (10)	0.0044 (10)	−0.0029 (8)	0.0000 (8)
N3	0.0452 (13)	0.0540 (14)	0.0608 (13)	−0.0057 (10)	0.0010 (11)	−0.0022 (11)
C1	0.0361 (13)	0.0385 (13)	0.0380 (11)	0.0045 (10)	0.0016 (9)	0.0033 (9)
C2	0.0410 (14)	0.0402 (14)	0.0445 (12)	0.0056 (11)	0.0023 (10)	0.0099 (10)
C3	0.0446 (14)	0.0381 (13)	0.0519 (13)	−0.0001 (11)	−0.0025 (11)	0.0006 (10)
C4	0.0396 (13)	0.0459 (14)	0.0384 (11)	0.0067 (11)	−0.0025 (10)	−0.0013 (10)
C5	0.0470 (15)	0.0489 (15)	0.0397 (12)	0.0013 (12)	0.0012 (10)	0.0103 (10)
C6	0.0425 (14)	0.0429 (14)	0.0454 (12)	−0.0016 (11)	0.0005 (10)	0.0076 (10)
C7	0.0354 (13)	0.0392 (14)	0.0397 (12)	0.0027 (11)	0.0042 (10)	0.0035 (10)
C8	0.0448 (15)	0.0495 (15)	0.0410 (12)	0.0057 (12)	−0.0006 (11)	0.0044 (11)
C9	0.0376 (13)	0.0427 (14)	0.0405 (12)	0.0040 (11)	0.0009 (10)	−0.0005 (10)
C10	0.0342 (13)	0.0404 (14)	0.0469 (13)	0.0040 (11)	0.0033 (10)	−0.0034 (10)
C11	0.0391 (14)	0.0488 (15)	0.0415 (12)	0.0077 (11)	−0.0009 (10)	−0.0001 (11)
C12	0.0382 (14)	0.0541 (16)	0.0456 (12)	0.0065 (12)	−0.0042 (10)	−0.0070 (11)
C13	0.0381 (14)	0.0425 (14)	0.0533 (13)	−0.0009 (11)	0.0007 (11)	−0.0044 (11)
C14	0.0444 (14)	0.0475 (15)	0.0453 (12)	0.0028 (12)	0.0007 (11)	0.0014 (11)
C15	0.0625 (18)	0.082 (2)	0.0418 (13)	0.0149 (15)	−0.0093 (12)	−0.0008 (12)

### Geometric parameters (Å, °)

**Table:**

O1—C4	1.353 (2)	C1—C7	1.461 (3)
O1—H1	0.8200	C2—C3	1.381 (3)
O2—C2	1.355 (2)	C3—C4	1.377 (3)
O2—H2	0.8200	C3—H3	0.9300
O3—C7	1.242 (2)	C4—C5	1.384 (3)
O4—C10	1.337 (2)	C5—C6	1.367 (3)
O4—H4	0.8200	C5—H5	0.9300
O5—N3	1.232 (2)	C6—H6	0.9300
O6—N3	1.216 (2)	C8—C9	1.447 (3)
O7—C11	1.366 (3)	C8—H8	0.9300
O7—C15	1.439 (2)	C9—C14	1.389 (3)
O8—H8A	0.855 (19)	C9—C10	1.401 (3)
O8—H8B	0.855 (19)	C10—C11	1.407 (3)
O9—H9A	0.857 (9)	C11—C12	1.368 (3)
O9—H9B	0.85 (2)	C12—C13	1.388 (3)
N1—C7	1.352 (3)	C12—H12	0.9300
N1—N2	1.370 (2)	C13—C14	1.368 (3)
N1—H1A	0.881 (10)	C14—H14	0.9300
N2—C8	1.274 (3)	C15—H15A	0.9600
N3—C13	1.455 (3)	C15—H15B	0.9600
C1—C2	1.401 (3)	C15—H15C	0.9600
C1—C6	1.402 (3)		
			
C4—O1—H1	109.5	O3—C7—N1	119.77 (18)
C2—O2—H2	109.5	O3—C7—C1	122.2 (2)
C10—O4—H4	109.5	N1—C7—C1	118.00 (19)
C11—O7—C15	117.01 (18)	N2—C8—C9	120.0 (2)
H8A—O8—H8B	104.2 (19)	N2—C8—H8	120.0
H9A—O9—H9B	106.7 (19)	C9—C8—H8	120.0
C7—N1—N2	117.96 (17)	C14—C9—C10	119.15 (19)
C7—N1—H1A	127.0 (17)	C14—C9—C8	120.0 (2)
N2—N1—H1A	115.1 (17)	C10—C9—C8	120.8 (2)
C8—N2—N1	118.57 (18)	O4—C10—C9	123.22 (18)
O6—N3—O5	122.4 (2)	O4—C10—C11	116.9 (2)
O6—N3—C13	118.6 (2)	C9—C10—C11	119.9 (2)
O5—N3—C13	118.98 (19)	O7—C11—C12	125.54 (19)
C2—C1—C6	117.07 (18)	O7—C11—C10	114.1 (2)
C2—C1—C7	118.85 (19)	C12—C11—C10	120.4 (2)
C6—C1—C7	124.1 (2)	C11—C12—C13	118.6 (2)
O2—C2—C3	117.5 (2)	C11—C12—H12	120.7
O2—C2—C1	121.57 (18)	C13—C12—H12	120.7
C3—C2—C1	120.9 (2)	C14—C13—C12	122.6 (2)
C4—C3—C2	120.3 (2)	C14—C13—N3	118.8 (2)
C4—C3—H3	119.9	C12—C13—N3	118.6 (2)
C2—C3—H3	119.9	C13—C14—C9	119.4 (2)
O1—C4—C3	117.5 (2)	C13—C14—H14	120.3
O1—C4—C5	122.60 (19)	C9—C14—H14	120.3
C3—C4—C5	119.93 (19)	O7—C15—H15A	109.5
C6—C5—C4	119.8 (2)	O7—C15—H15B	109.5
C6—C5—H5	120.1	H15A—C15—H15B	109.5
C4—C5—H5	120.1	O7—C15—H15C	109.5
C5—C6—C1	122.0 (2)	H15A—C15—H15C	109.5
C5—C6—H6	119.0	H15B—C15—H15C	109.5
C1—C6—H6	119.0		

### Hydrogen-bond geometry (Å, °)

**Table:**

*D*—H···*A*	*D*—H	H···*A*	*D*···*A*	*D*—H···*A*
O8—H8B···O4^i^	0.86 (2)	2.39 (2)	2.953 (2)	124 (2)
O8—H8B···O7^i^	0.86 (2)	2.17 (1)	3.001 (3)	163 (2)
O9—H9B···O3^ii^	0.85 (2)	2.19 (1)	3.032 (2)	170 (3)
O9—H9A···O2^iii^	0.86 (1)	1.98 (1)	2.840 (2)	176 (2)
O8—H8A···O9^iv^	0.86 (2)	1.93 (1)	2.786 (2)	176 (2)
N1—H1A···O5^v^	0.88 (1)	2.55 (2)	3.183 (3)	130 (2)
N1—H1A···O8^ii^	0.88 (1)	2.44 (2)	3.195 (3)	143 (2)
O4—H4···N2	0.82	1.85	2.569 (2)	146
O2—H2···O3	0.82	1.80	2.526 (2)	147
O1—H1···O8^vi^	0.82	1.91	2.718 (2)	169

Symmetry codes: (i) *x*+1, *y*, *z*; (ii) −*x*+1, −*y*+1, −*z*+1; (iii) *x*, *y*−1, *z*; (iv) −*x*+2, −*y*+1, −*z*+1; (v) −*x*, −*y*, −*z*+1; (vi) *x*, *y*, *z*+1.
